# The Scholarship Circle: an introduction to writing for publication for nursing faculty

**DOI:** 10.5195/jmla.2020.685

**Published:** 2020-01-01

**Authors:** Kerry Dhakal, Joni Tornwall

**Affiliations:** Health Sciences Library, Ohio State University, Columbus, OH, dhakal.9@osu.edu; College of Nursing, Ohio State University, Columbus, OH, tornwall.2@osu.edu

## Abstract

**Background:**

This case report describes a collaborative effort between a health sciences librarian and an instructional designer to create and implement a writing professional development experience called the Scholarship Circle. It was aimed at increasing scholarly productivity by junior and nontenure-track faculty in a college of nursing.

**Case Presentation:**

The Scholarship Circle activities were carried out in a synchronous and an asynchronous online environment over ten weeks and included weekly lectures from nurse-scholars, discussions and peer reviews, and writing support from the librarian. The Scholarship Circle designers surveyed participants before and after the course to explore faculty perceptions and conducted a bibliographic analysis to gauge increases in scholarly productivity.

**Conclusions:**

While both tenure-track and nontenure-track faculty perceived lack of time as a significant barrier to publication, only nontenure-track faculty perceived lack of writing experience and getting started as significant obstacles. In the two years following the Scholarship Circle, faculty with doctor of philosophy and doctor of education degrees produced the greatest number of scholarly publications, whereas faculty with other degrees demonstrated a modest increase in scholarship. Online writing support programs have the potential to positively impact scholarly productivity for junior and nontenure-track faculty, especially if they emphasize time management for writing, confidence-building strategies, and a flexible format that allows peer review and collaboration as well as participation by seasoned scholars and remote participants. Partnership between health sciences librarians and instructional designers is key to the successful design and implementation of writing support programs.

## BACKGROUND

Writing for publication is one of the primary expectations for academic faculty in most universities and colleges. However, many faculty members who have degrees other than a doctor of philosophy degree (PhD) and doctor of education degree (EdD) have not had sufficient instruction on how to approach the process of writing scholarly manuscripts that are intended for publication [1–9]. According to Gazza and Hunker, many nursing education programs do not include clear paths for nurse graduates to develop writing skills and competencies [1], in particular where scholarly writing is concerned [8]. There are also many perceived situational and personal barriers for clinicians to publish, including lack of time, unfamiliarity with the publishing process, and concern about their own ability to write well [3, 7]. Some health care systems and organizations have tried to address these situational and personal barriers by providing individual consultations, mentoring, role modeling, writing support groups, writing courses, and other writing support activities for their staff members [3, 7].

Positive outcomes from efforts to facilitate writing and publication are well documented in nursing and library science literature. These benefits include increased scholarly productivity as well as ancillary benefits that are not directly related to publishing, such as development of sustained collegial relationships and team building [10], enhanced skills in poster preparation and research design [11], and professional networking and academic promotion [12]. Furthermore, when it is approached in a structured and supported manner, support for scholarly productivity for clinical staff and junior faculty promotes professional growth and improves patient care outcomes [6, 7].

In an effort to encourage junior and nontenure-track nursing faculty to consider writing for publication and participate more fully in scholarly activities, the Health Sciences Library and the College of Nursing at the Ohio State University collaborated on developing and implementing an online professional development course in the summer of 2016 called the Scholarship Circle. The course also provided an opportunity to teach participants about human and material resources that were available to faculty who were writing for publication, including library resources and librarian services, based on a collaborative model used regularly by liaison librarians in health sciences libraries [13–15]. This report describes the Scholarship Circle course, data collected from surveys, and results from a bibliographic analysis of participants’ publications.

## CASE PRESENTATION

The Ohio State University’s College of Nursing enrolls more than 1,800 students in bachelor’s, master’s, and doctoral nursing programs and employs about 150 faculty, with over half holding a nontenure-track faculty position. All faculty are expected to engage in evidence-based practice, and scholarly productivity is also strongly encouraged. Publication bears significant weight on advancement prospects for all faculty at this public university with a strong research focus, and academic administrators want to support all nursing faculty in broadening their skills and capacity to publish scholarly works. The idea for the Scholarship Circle emerged as the College of Nursing and Health Sciences Library faculty were trying to identify the best approach to offering consultation and education about scholarship to College of Nursing faculty.

### Course design

The Scholarship Circle course was a blend of synchronous and asynchronous activities, housed primarily in the university’s Canvas learning management system. The authors developed and facilitated the course in our roles as the College of Nursing’s instructional designer and one of the Health Sciences Library’s research and education librarians. We chose an online format for course content delivery to make the Scholarship Circle available to the large number of nine-month faculty who were not on campus during the summer but wanted to pursue goals related to scholarship. Faculty participants were expected but not required to complete the asynchronous portions of each weekly module before attending the synchronous sessions.

The asynchronous, online portion of the Scholarship Circle course was based on an open, online course called Writing for Professional Journals, developed by Patricia Morton [16]. To accompany and support the course content, we created a LibGuide (supplemental Appendix A) that provided resources and information about conducting literature reviews, citation management, journal selection, and use of journal and citation tools, based on evidence in the literature showing that sharing additional subject-specific information in an organized way can be helpful to course participants [17].

We offered the synchronous, weekly sessions over a ten-week period. The sessions consisted of nine live, thirty-minute lectures from featured nurse-scholars with notable accomplishments in publishing, consulting, or editing in nursing literature and one lecture from the librarian who co-facilitated the course. The online format of the course provided flexibility in selecting nurse-scholar lecturers from across the country with various types of expertise in publishing. Each lecture concluded with fifteen to twenty minutes of interaction between the participants and the featured author or editor. Topics included how to choose a journal, how to get started and avoid procrastination, how to handle rejection and negative feedback, and how to use tools and databases to select journals for publication and identify journal impact factors.

We reserved the second half of the synchronous sessions for collaborative work on manuscripts or peer review, group discussion related to specific issues with manuscripts or the publishing process, and one-on-one consultations with the course facilitators or in-house experts in nurse scholarship. For example, a professor and seasoned author from the college shared her experiences with manuscript review, explaining that grappling with rejection and criticism is a normal part of writing for publication that one must learn to deal with objectively. All participants attended the synchronous sessions virtually via Adobe Connect, a mobile web conferencing application, even if they were on campus, and most of the collaboration and sharing occurred in the virtual meeting space.

We strongly encouraged participants to complete the asynchronous course content before attending the synchronous sessions, and we estimated that doing so would take about one hour each week. The asynchronous content prompted participants to reflect on barriers to writing and how to overcome them, to write aims for a manuscript and begin the writing process, and to work on different sections of their papers (e.g., introduction, methods, results). While most participants started the first week by completing the online activities, progressively fewer participants completed the online activities as the ten weeks passed, citing lack of time as the reason. Some faculty expressed a desire to write collaboratively with other faculty members during the sessions, but by the end of the ten-week course, no new authorship collaborations had been formed.

### Participants

The Scholarship Circle course was open to any faculty member; however, the primary goal of this course was to increase scholarly productivity for junior and nontenure-track nursing faculty. Demographic data suggested that the course reached the intended audience, as twenty-two of the thirty participants were junior or nontenure-track faculty.

Because College of Nursing faculty have different ranks (i.e., assistant, associate, or full professor; clinical or research faculty) and status types (i.e., tenure-track or nontenure-track), participants were asked to identify themselves by their status on a pre-course survey. They were also asked to select their highest level of education as an indicator of how much previous formal training they may have had in writing for publication.

**Figure 1:**
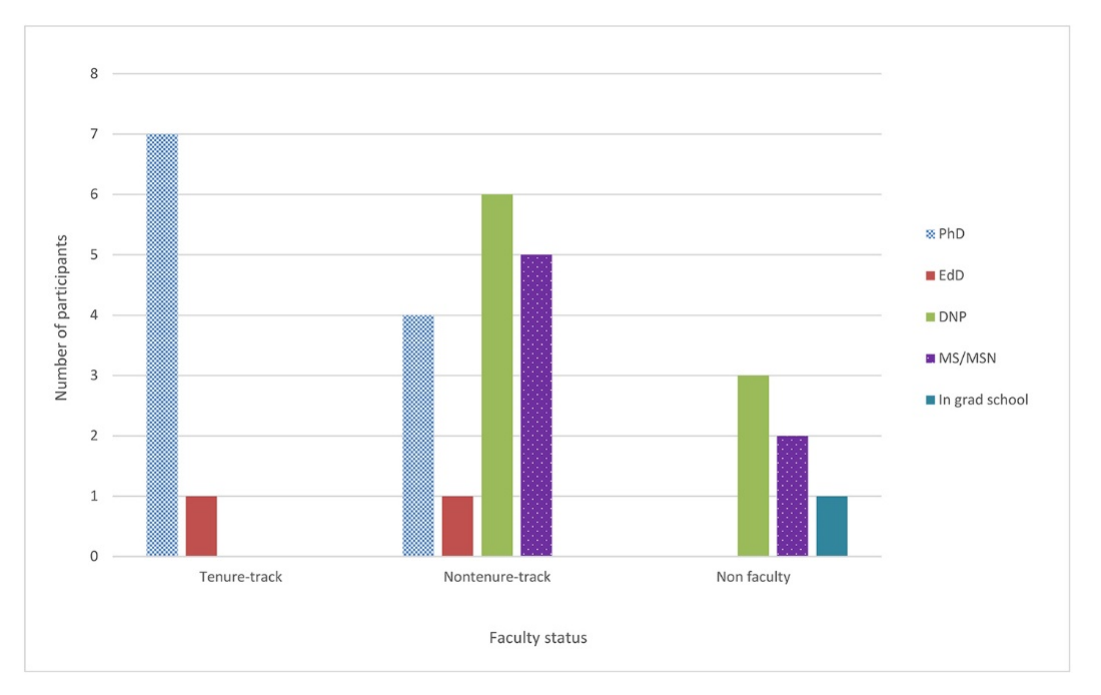
Degree earned by faculty status

A total of 30 faculty participated in the course (Figure 1). The majority of participants (n=16) were nontenure-track faculty members with a clinical, teaching, or research focus. Eight were on the tenure track, 2 were tenured, and 6 were not yet tenured. Five non-faculty nurses from the Ohio State University Wexner Medical Center and 1 graduate nursing student also participated. This study was determined to be exempt from review by the Ohio State University’s Institutional Review Board.

### Data collection

Course participants were invited to complete a survey before the course began, a few days after the course ended, six months after the course, and one year after the course to obtain a complete picture of whether faculty perspectives on writing changed and whether article completion and submission increased over a year’s time (supplemental Appendix B). The online survey consisted of multiple-choice and open-ended questions regarding the participants’ work experience and context, publishing history, perceptions regarding barriers to publishing, journal selection, and the impact of nursing research as well as what they expected to gain from participating in the writing course.

**Figure 2:**
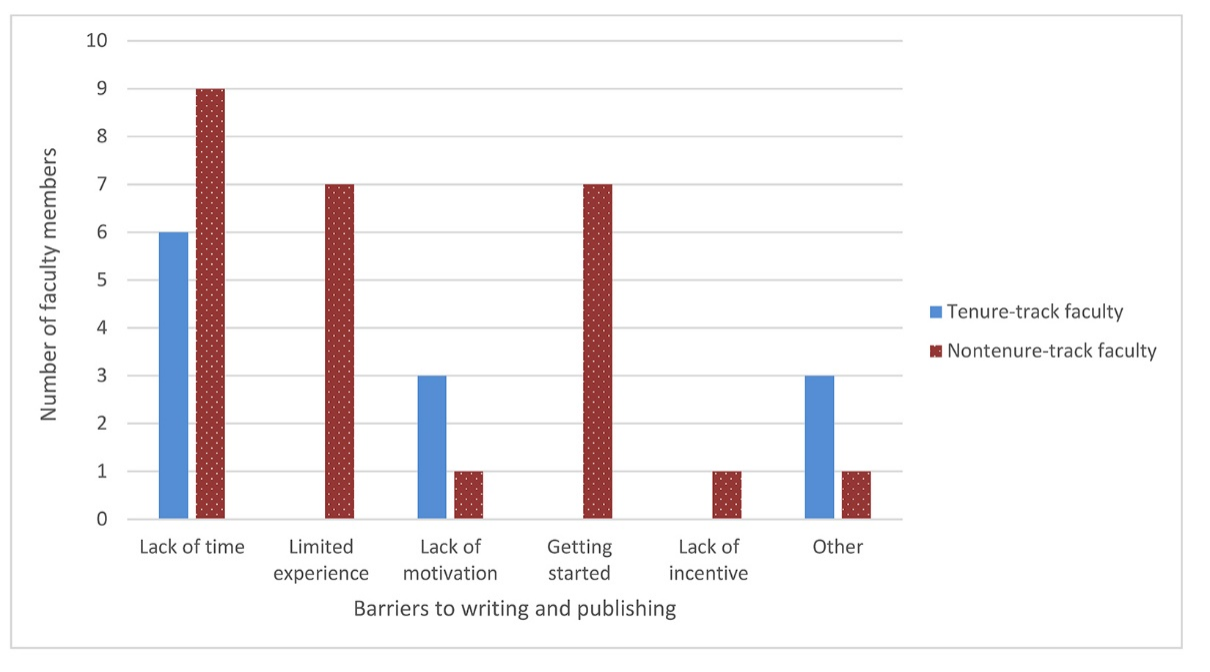
Barriers to writing for publication before participation in the Scholarship Circle by faculty status

Due to low response rates, the six-month and one-year surveys did not yield useful information. However, from the pre- and immediate post-course survey data, two main issues emerged as significant barriers that participants faced when thinking about writing: lack of time and lack of experience. The full list of barriers identified by the eighteen participants who responded to the pre-course survey is shown in Figure 2.

Additional barriers that were described in short-answer responses by tenure-track faculty included difficulty motivating collaborators, slowness of coauthor responses, and procrastination. Other barriers listed by nontenure-track faculty members included choosing a topic, coordinating the timing of writing tasks with coauthors, and a comment that “writing is a difficult process.” Of the six participants who responded to the post-course survey, three continued to perceive lack of time to write and limited experience as significant barriers to writing, and two stated that there were other barriers, including journal publishers’ slow responses to questions. One participant responded that they were not motivated and had no self-discipline to start writing. None of these participants provided information about their faculty status.

**Figure 3:**
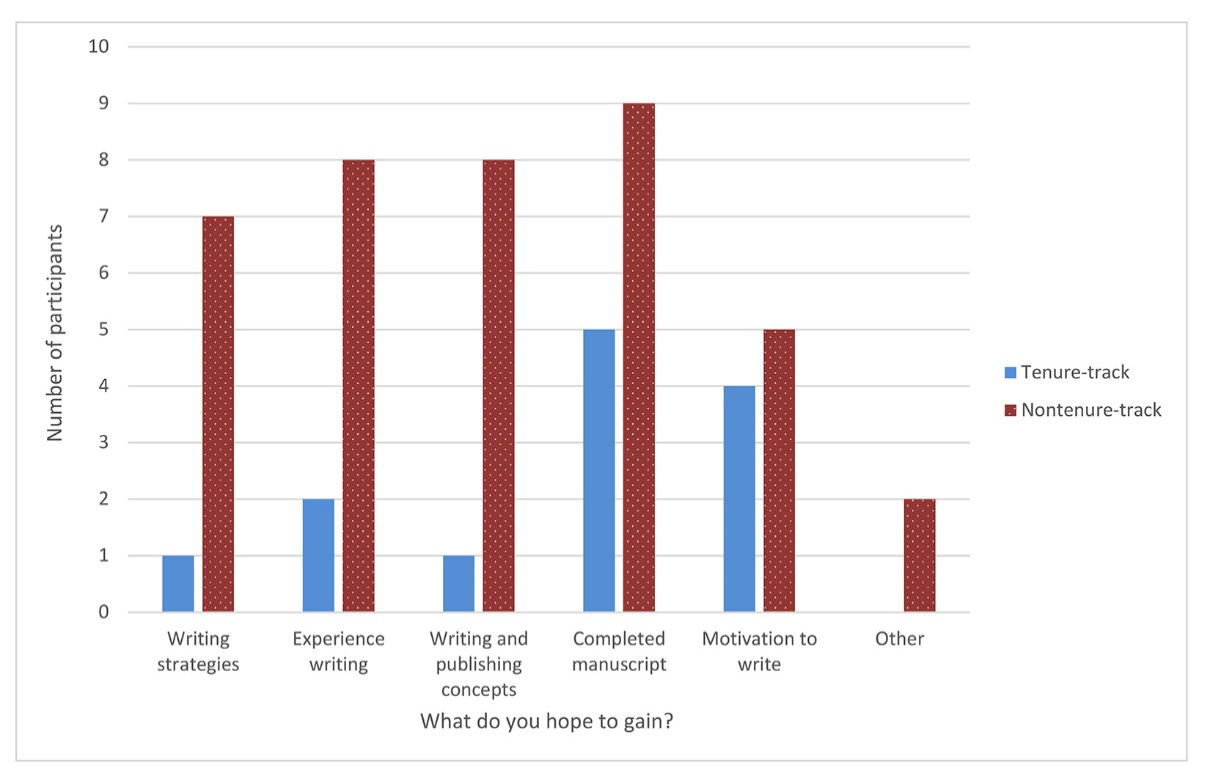
What do you hope you will gain by participating in the Scholarship Circle?

When participants were asked what they hoped to gain from participating in the Scholarship Circle before it began and what they actually gained after the Scholarship Circle ended, results varied. Both nontenure-track and tenure-track faculty members were hoping to complete a manuscript by the end of the Scholarship Circle course (Figure 3). Although participants indicated that they gained experience in the Scholarship Circle (yet still perceived lack of experience as a barrier), none of the participants achieved their goal of writing a manuscript before the Scholarship Circle sessions ended. However, one tenure-track faculty member submitted a manuscript approximately two months after the Scholarship Circle.

The tenure-track faculty members seemed less interested overall than nontenure-track faculty in gaining experience or learning strategies and concepts about writing and publishing, but both groups were similarly interested in gaining motivation to write as a result of their participation in the course. Two nontenure-track faculty members who responded in the “Other” category stated that they hoped to gain knowledge about how to choose a journal for publication and to have a forum where they could prioritize writing and making time to write. Of the six participants who responded to this question in the post-course survey, five said they gained experience with strategies and tools for writing and publishing articles, four learned new concepts to help them think about writing or publishing, and two learned about strategies for finding time and motivation for writing and publishing an article (this question allowed multiple answers).

### Bibliographic analysis results

To address some of the gaps in data due to low participation rates on the post-course surveys, we conducted a bibliographic analysis to compare the number of publications participants had in the two and a half years before the Scholarship Circle course and the two and a half years after the course was completed. The search included publications in the health sciences and nursing education literature indexed in PubMed, CINAHL, ERIC, and Scopus between January 2014 and July 2016 and between August 2016 and December 2018. We searched by author name and university affiliation to find articles authored by participants. Taking into consideration that a few participants were not at the Ohio State University in the years preceding the Scholarship Circle, the search included publications from other universities. After compiling the number of articles published by each participant, we examined whether level of education or faculty status influenced the number of publications.

Scholarship Circle participants published 124 scholarly works between 2014 and 2018, with 38 publications between January 2014 and July 2016 and 86 between August 2016 and December 2018 (Table 1). Faculty participants, regardless of tenure-track status, published more articles both before and after the Scholarship Circle course than non-faculty participants. Overall, tenure-track faculty published more than nontenure-track faculty participants. The 7 tenure-track faculty members with a PhD published almost double the amount of nontenure-track faculty members with a PhD in 2014–2016 (21 versus 11 articles) and double the number of articles in 2016–2018 (42 versus 21 articles). The 9 participants with a doctor of nursing practice (DNP) degree published a total of 3 articles before the course and 9 articles afterward. However, there were also 4 participants with DNPs who did not publish either before or after participating in the Scholarship Circle. The 7 participants with a master’s degree in a nursing discipline had not published any articles prior to participating in the Scholarship Circle, but 2 published articles after the Scholarship Circle. Five participants with master’s degrees did not publish before or after the course.

**Table 1:** Total number of articles published by faculty status and level of education (before and after the course)

Participant type*	n	Number of published articles (2014–2016)			Total	Number of published articles (2016–2018)			Total	Cumulative total
	
		PhD	EdD	Other*		PhD	EdD	Other*		
Tenure-track faculty	8	21	1	0	22	42	3	0	45	67
Nontenure-track faculty	16	11	1	1	13	21	10	0	31	44
Non-faculty	6	0	0	3	3	0	0	10	10	13
Total					38				86	**124**

* Other degree types include doctor of nursing practice (DNP) or master’s of science/master’s of science in nursing (MS/MSN) degree.

A comparison of publishing rates between faculty with PhD or EdD degrees and faculty with other types of degrees revealed significant differences before and after the Scholarship Circle sessions. An independent samples *t*-test showed that faculty with a PhD or EdD published significantly more before the Scholarship Circle (mean [M]=2.62, standard deviation [SD]=1.45) than faculty with DNP or master’s degrees (M=1.06, SD=1.25; *t*(15)=5.62, *p*<0.001). This pattern persisted after the Scholarship Circle, with faculty with a PhD or EdD (M=5.23, SD=4.23) publishing more than faculty with a DNP or master’s degree (M=1.06, SD=1.25; *t*(14)=3.45, *p*=0.004). Comparing within-subjects differences, a paired samples *t*-test showed that faculty with a PhD or EdD published more after the Scholarship Circle than before (*t*(12)=2.68, *p*=0.020). Similarly, faculty with a DNP or master’s degree published more articles after the Scholarship Circle than before (*t*(16)=3.85, *p*=0.001).

## DISCUSSION

The findings from this study suggest that nursing faculty members, whether on the tenure track or not, prefer synchronous online coursework to asynchronous coursework when they are learning about the process of writing for publication. We also found that faculty with degrees other than PhDs and EdDs can benefit from more hands-on support when they are working through the process of considering publication and writing manuscripts. Writing support could come from nurse-scholars who are willing to share their knowledge through courses like the Scholarship Circle or writing groups and other on-campus service providers who assist faculty and non-faculty with writing and publishing activities, including health sciences librarians. Our results further suggest that finding time to write and lack of experience in the writing process may be barriers to successful publication.

### Synchronous instruction preferred

According to course participation statistics and interactive observations, participants attended the weekly synchronous course meetings online. However, it was difficult to persuade participants to take time to view the videos and complete the exercises before attending the synchronous sessions, even though the asynchronous materials consisted of high-quality content and easily understandable descriptions of the steps of the publishing process. Mears and Blake suggest that encouraging participants, sending reminder email messages, and offering incentives for completing the activities of online courses could motivate participants to engage in the asynchronous portion of such courses [18].

### Time and motivation

Rees and colleagues note that lack of time is one of the most common barriers to writing for publication [5]. Participants of the Scholarship Circle likewise responded in the pre-course survey that lack of time was a barrier to writing for publication and that they hoped to gain motivation to write and complete a manuscript by the end of the course. Even though no participants completed a manuscript, many regularly made time to attend the synchronous online weekly meetings of the course and were motivated to engage in course activities and discussions. Two years after the Scholarship Circle course, it was evident that some participants made time and gained motivation to write for publication, particularly tenure-track faculty members with a PhD, nontenure-track faculty members with a PhD or EdD, and non-faculty members with a DNP or master of science/master of science in nursing (MS/MSN) degree. We cannot say with certainty that there is a causal relationship between participation in the Scholarship Circle and the number of articles published by faculty and non-faculty members with different levels of education, but it appears that faculty without a PhD or EdD may need additional support. Further research is needed to determine how perceptions of lack of time and motivation impact nursing faculty members when they are writing for publication.

### Collegial writing support

Literature suggests that developing a culture of scholarship and providing access to and encouragement from academic faculty and professional nurses who write and publish regularly can be helpful [3, 5, 7]. The Scholarship Circle offered direct weekly access to seasoned nursing writers who shared their experiences in the writing and publishing process, including providing strategies on how to get started in writing and being prepared to receive constructive feedback through the peer-review process. The asynchronous content also provided guidance from experienced nursing faculty authors even though it was mostly not used by Scholarship Circle participants.

We also promoted the services and resources that exist through the Health Sciences Library to support nursing faculty in the scholarly writing process by having a research and education librarian, who is also the nursing liaison librarian, partner in teaching and facilitating the Scholarship Circle. As von Isenberg and colleagues note, librarians are supportive collaborators in the writing process since they already provide guidance on many aspects of the writing and publishing process, such as developing search strategies, conducting literature reviews, finding and selecting journals for publication, and using reference management tools [13]. Scholarship Circle participants consulted with the librarian during and after the course with requests for assistance with these aspects of writing. This helped establish a relationship of trust that has persisted to the present time, generating requests for assistance from faculty with writing and publishing manuscripts.

Limitations of the study included a low response rate for the post-course surveys. The responses provided might not reflect the thinking of the majority of faculty and non-faculty regarding barriers to writing and gains that they had hoped to make during the course. Also, the anonymity of the pre- and post-course surveys made it impossible to pair individual responses with the level of education.

## CONCLUSION

Based on the results of this study, a key factor in scholarship productivity appears to be degree preparation, with nursing faculty who have a PhD or EdD producing significantly more published articles than nursing faculty with DNPs or master’s degrees. Additionally, non-faculty participants with a DNP or MS/MSN degree produced more published articles after the course than faculty with a DNP or MS/MSN degree. A professional development program specifically designed to positively influence scholarship productivity for nontenure-track nursing faculty with clinically focused degrees appeared to have worthwhile scholarship outcomes for all nursing faculty, though it did not equalize publishing rates between nurses with different degree types when scholarship productivity was measured in the two and a half years after the program. Finally, collaboration between librarians and instructional design staff in nursing education can facilitate development of engaging and flexible professional development that supports positive attitudes and intentions toward professional writing for publication.

## Supplemental Files

Appendix A Scholarship Circle writing course LibGuide screenshot

Appendix B Survey

## 

**Kerry Dhakal,** dhakal.9@osu.edu, http://orcid.org/0000-0001-7782-5922, Health Sciences Library, Ohio State University, Columbus, OH

**Joni Tornwall,** tornwall.2@osu.edu, https://orcid.org/0000-0001-8479-8352, College of Nursing, Ohio State University, Columbus, OH
