# Supplementary material for: The Scholarship Circle: an introduction to writing for publication for nursing faculty
Source: J Med Libr Assoc. 2020 Jan 1;108(1):98–105. doi: 10.5195/jmla.2020.685 (PMC6920005; doi:10.5195/jmla.2020.685)
Supplement: Appendix A [file jmla-108-98-s001.pdf]

JMLA Journal of the Medical Library Association

Supplemental content to
J Med Libr Assoc. Jan;108(1):dx.doi.org/10.5195/jmla.2020.685
www.jmla.mlanet.org
© Dhakal, Tornwall 2020

The Scholarship Circle: an introduction to writing for publication for nursing faculty

Kerry Dhakal; Joni Tornwall

APPENDIX A

Scholarship Circle writing course LibGuide screenshot

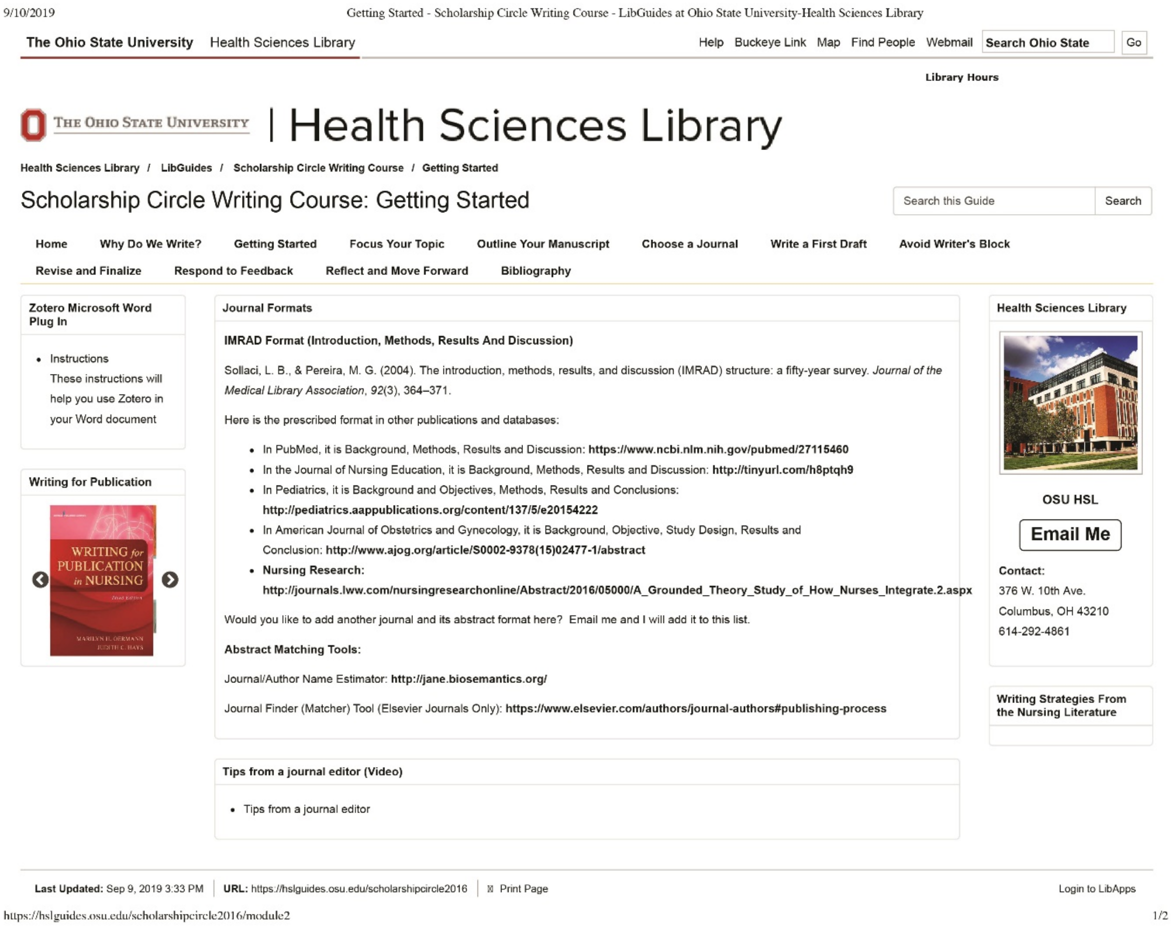

9/10/2019 Getting Started - Scholarship Circle Writing Course - LibGuides at Ohio State University-Health Sciences Library

The Ohio State University Health Sciences Library Help Buckeye Link Map Find People Webmail Search Ohio State Go

Library Hours

THE OHIO STATE UNIVERSITY | Health Sciences Library

Health Sciences Library / LibGuides / Scholarship Circle Writing Course / Getting Started

Scholarship Circle Writing Course: Getting Started

Search this Guide Search

Home | Why Do We Write? | Getting Started | Focus Your Topic | Outline Your Manuscript | Choose a Journal | Write a First Draft | Avoid Writer's Block | Revise and Finalize | Respond to Feedback | Reflect and Move Forward | Bibliography

Zotero Microsoft Word Plug In

- Instructions
  These instructions will help you use Zotero in your Word document

Writing for Publication

Journal Formats

IMRAD Format (Introduction, Methods, Results And Discussion)

Sollaci, L. B., & Pereira, M. G. (2004). The introduction, methods, results, and discussion (IMRAD) structure: a fifty-year survey. *Journal of the Medical Library Association*, *92*(3), 364–371.

Here is the prescribed format in other publications and databases:

- In PubMed, it is Background, Methods, Results and Discussion: **https://www.ncbi.nlm.nih.gov/pubmed/27115460**
- In the Journal of Nursing Education, it is Background, Methods, Results and Discussion: **http://tinyurl.com/h8ptqh9**
- In Pediatrics, it is Background and Objectives, Methods, Results and Conclusions: **http://pediatrics.aappublications.org/content/137/5/e20154222**
- In American Journal of Obstetrics and Gynecology, it is Background, Objective, Study Design, Results and Conclusion: **http://www.ajog.org/article/S0002-9378(15)02477-1/abstract**
- **Nursing Research: http://journals.lww.com/nursingresearchonline/Abstract/2016/05000/A_Grounded_Theory_Study_of_How_Nurses_Integrate.2.aspx**

Would you like to add another journal and its abstract format here? Email me and I will add it to this list.

Abstract Matching Tools:

Journal/Author Name Estimator: **http://jane.biosemantics.org/**

Journal Finder (Matcher) Tool (Elsevier Journals Only): **https://www.elsevier.com/authors/journal-authors#publishing-process**

Tips from a journal editor (Video)

- Tips from a journal editor

Health Sciences Library

OSU HSL

Email Me

Contact:
376 W. 10th Ave.
Columbus, OH 43210
614-292-4861

Writing Strategies From the Nursing Literature

Last Updated: Sep 9, 2019 3:33 PM | **URL:** https://hslguides.osu.edu/scholarshipcircle2016 | Print Page Login to LibApps

https://hslguides.osu.edu/scholarshipcircle2016/module2 1/2
