# Supplementary material for: The Scholarship Circle: an introduction to writing for publication for nursing faculty
Source: J Med Libr Assoc. 2020 Jan 1;108(1):98–105. doi: 10.5195/jmla.2020.685 (PMC6920005; doi:10.5195/jmla.2020.685)
Supplement: Appendix B [file jmla-108-98-s002.pdf]

JMLA Journal of the Medical Library Association

SC

Supplemental content to
J Med Libr Assoc. Jan;108(1):dx.doi.org/10.5195/jmla.2020.685
www.jmla.mlanet.org
© Dhakal, Tornwall 2020

The Scholarship Circle: an introduction to writing for publication for nursing faculty

Kerry Dhakal; Joni Tornwall

APPENDIX B

Survey

Intro block

Consent to participate in research

Study Title: Scholarly Publishing in Nursing and Measuring Its Impact

Read the following information and select "I agree" on the bottom of this page if you would like to participate.

This is a consent form for research participation.

It contains important information about this study and what to expect if you decide to participate.

Your participation is voluntary.

You are invited to participate in a research study about how a writing course can impact the scholarly productivity of nurses and how nurses who are interested in publishing scholarly articles think about the concept of research impact. Your participation in this research study is voluntary, and you may choose not to participate, or to stop participating, at any time. You may skip any question for any reason. You will not be penalized for not participating or stopping your participation. Risks to you are no more than minimal.

Feel free to ask questions before making your decision about whether or not to participate. If you decide to participate in the survey, you will provide your consent by checking a box at the bottom of this page acknowledging that you have read this information and by submitting your survey responses. No signature or hard copy of this consent is necessary.

What the study is about:

> The purpose of this study is to learn if a writing course can positively impact the scholarly productivity of nurses and how nurses who are interested in publishing scholarly articles think about the concept of research impact. The information collected in this study will inform future conversations about how nursing scholars think about the concept of research impact and the context of the larger discipline-specific perception of the concept of research impact.

We are collecting information for two purposes: (1) to determine the impact of a writing course on scholarly productivity, and (2) to explore the considerations nursing scholars take when deciding where to publish scholarly articles in the field of nursing, with a particular focus on how nursing scholars define and measure research impact.

What we will ask you to do

If you agree to participate in this study, you will be asked to complete a survey at four points:

1. near the beginning of the writing course,
2. at the end of the writing course,

SC
Supplemental content to
J Med Libr Assoc. Jan;108(1):dx.doi.org/10.5195/jmla.2020.685
www.jmla.mlanet.org
© Dhakal, Tornwall 2020

3. 6 months after the end of the writing course, and
4. 12 months after the end of the writing course.

The surveys will ask some questions about your nursing specialty, experience publishing journal articles, barriers you have faced doing so, and your perceptions of research impact in nursing. Once you have consented to participate in a survey, the survey should take five to ten minutes to complete. You may skip any question you feel uncomfortable answering. If you decide to stop participating in the project before you submit your results, there will be no penalty to you. The researchers will be responsible for collecting the data for this study. They will maintain the anonymity of your participation. Please note that once you provide your consent and submit your responses to the online survey, your submission cannot be withdrawn unless you provide the researchers with your unique code that you create. Your data will be submitted anonymously and cannot be traced back to you without your code.

You will also have the opportunity to participate in a focus group at the end of the writing course, as the last session of the course. In the focus group, one of the researchers will facilitate a discussion of your perceptions of the effectiveness of the writing course and your perspectives on the concept of research impact in nursing, as well as other observations at the group level. Your consent for participation in the focus group will be obtained separately from your consent for the surveys.

If you participate in the focus group, the focus group will take approximately one hour. You may choose to participate in any, all, or none of the survey and focus group opportunities. The researchers hope to obtain a clear picture of the effect of full participation, but lack of participation in one survey or focus group opportunity will not invalidate your responses on the other opportunities. Therefore, your participation, even if it is partial, is still valuable.

Risks and benefits

We do not anticipate any risks to you participating in this study other than those encountered in day-to-day life. There is no compensation for this survey. There are benefits to you in that the information we gather in the surveys will advance the understanding of the effectiveness of the writing course on scholarly productivity and the perspective of nurse-scholars on research impact in nursing. Your participation in the focus group and the surveys will contribute to public knowledge and the ongoing conversation in the nursing field about scholarly publishing and the concept of research impact. Participation in the surveys and focus groups also provides you with an opportunity to reflect on your own perspectives and growth as a scholar over the yearlong project as well as an opportunity to hear the perspectives of other nurse-scholars in the focus group.

Confidentiality

Your answers will be confidential. You will be identified only by a code you create from a combination of numbers familiar to you. The purpose of the code is to match your responses over the four surveys and the focus group. The records of this study will be kept private. In any report we make public, we will not include information that will make it possible to identify you.

All responses will be used for scholarly purposes. We will work to make sure that no one sees your survey responses without approval. However, because we are using the Internet, there is a small chance that someone could access your online responses without permission. In some cases, this information could be used to identify you. Your data will be protected with a code you create to reduce risk that other people can view the responses.

SC
Supplemental content to
J Med Libr Assoc. Jan;108(1):dx.doi.org/10.5195/jmla.2020.685
www.jmla.mlanet.org
© Dhakal, Tornwall 2020

Participant rights

You may refuse to participate in this study without penalty or loss of benefits to which you are otherwise entitled. If you are an employee at the university, your decision and responses will not affect your employment status.

If you have questions: Please contact the researchers with any questions. For questions about your rights as a participant in this study or to discuss other study-related concerns or complaints with someone who is not part of the research team, you may contact the University Office of Responsible Research Practices at 800.678.6251.

Consent block

By selecting "I agree" below, I acknowledge that I have read this consent form and am aware that I am being asked to participate in a research study by completing a survey that will begin on the next page. I have had the opportunity to ask questions about this research study and have had them answered to my satisfaction.

I agree
I prefer not to participate

Code creation

In this step, you will create a unique code by which your survey responses will be matched up over the four different opportunities you have to complete the Scholarship Circle surveys.

In the field below, type the last two digits of your home address. For example, if you live at 1234 Memory Lane, you would type 34.

In the field below, type the last two digits of your cell phone number. For example, if your cell phone number is 987.654.3210, you would type 10.

In the field below, type the numbers that represent the month of your birth. For example, if you were born in February, you would type 02.

Your code for this study is [######].

You will be prompted to enter your code with the same prompts (address, cell phone number, and birth month) next time you complete a Scholarship Circle survey. If you are not sure you will remember the numbers you entered when you are prompted on the next survey, please write down this code and save it so you can enter it on your next survey submission at the end of the Scholarship Circle experience.

Professional title

Which of the following best describes your primary professional role? Please choose only one.

DNP at this university medical center
Faculty or staff at this university college

Which of the following best describes your title at the college?

Associated faculty
Tenure tracking or tenured clinical track faculty
Research faculty

SC
Supplemental content to
J Med Libr Assoc. Jan;108(1):dx.doi.org/10.5195/jmla.2020.685
www.jmla.mlanet.org
© Dhakal, Tornwall 2020

Which of the following associated faculty descriptors best describes your title at the college?

- Instructor of clinical practice (master's of science [MS]–prepared)
- Assistant professor of clinical practice (doctor of nursing practice [DNP]–- or doctorate [PhD]-prepared)
- I'm not sure right now

Which of the following tenure tracking or tenured descriptors best describes your title at the college?

- Associate professor
- Assistant professor
- Professor
- I'm not sure right now

Which of the following clinical track faculty descriptors best describes your title at the college?

- Associate assistant
- Professor of clinical nursing instructor
- I'm not sure right now

Professional context and experience

What is your principal nursing specialty? Please choose only one.

- Community/public health
- Critical care
- Emergency or trauma
- Family practice
- Geriatric
- Medical-surgical
- Neonatal
- Intensive care
- Obstetrics
- Oncology
- Pediatric
- Psych/mental health
- Women's health
- Nursing research
- Other

How many years have you practiced as a nurse in an academic setting (at this university or any other academically affiliated institution)?

- Less than 1 year
- 1–3 years
- 4–6 years
- 7–15 years
- More than 15 years

Publishing history

Have you ever written any journal articles that have been published?

- Yes
- No

SC
Supplemental content to
J Med Libr Assoc. Jan;108(1):dx.doi.org/10.5195/jmla.2020.685
www.jmla.mlanet.org
© Dhakal, Tornwall 2020

During your nursing/academic career, how many journal articles have you published? Please count only those articles that are published in a journal or accepted and scheduled to be published that count toward your professional promotion and tenure requirements.

- 1–2
- 3–4
- 5 or more

What kinds of works have you published? For this question, consider all kinds of articles or works you have created that have been made available broadly, and use the "other" field to further describe your work, if you wish.

- Research article based on collected data
- Integrative/systematic review
- Practice article for the clinical setting
- Practice article for education or the classroom setting
- Other

Current stage of understanding of metrics

Do you have a target journal in mind to which you would like to submit an article?

- Yes
- No

What is the name of the journal to which you would like to submit an article? If you have more than one journal in mind, please feel free to list multiple journals. Our librarian will take this information into consideration as she is preparing her Scholarship Circle presentation and when she assists college faculty in their publishing endeavors.

What criteria do you or would you use to select a journal(s) for submission of the articles you write? Click and drag each item to rank it in order of importance (1–9), where 1 is the most important and at the top of the list and 9 is the least important and at the bottom of the list.

- Journal impact factor
- Accessibility (open access)
- Types of articles covered by the journal
- Author rights (intellectual property)
- Size of readership
- Time to publication
- Journal is peer reviewed
- Quality of accepted articles and authors
- Audience

Of the following journal impact metrics, **which ones could you define with reasonable confidence in a sentence or two if a colleague were to ask you what they are in a casual conversation?**

There is absolutely no expectation that you are familiar with any of these metrics. Many of them will be covered in the Scholarship Circle by our librarian. The purpose of this question is only to gauge familiarity with the many ways research impact is currently measured.

- Journal impact factor
- Citation counts
- H-index
- Altmetrics

SC
Supplemental content to
J Med Libr Assoc. Jan;108(1):dx.doi.org/10.5195/jmla.2020.685
www.jmla.mlanet.org
© Dhakal, Tornwall 2020

G-index
Eigenfactor
I am not familiar with any of these metrics yet.

In your opinion, are the typical measures of research impact (journal impact factor, h-index, citation count, etc.) a good reflection of the real impact of nursing research? That is, do the typical measures of impact listed in the previous question provide a good measurement of the real impact in the discipline of nursing? Click and drag the sliding bar to indicate your perception.
Very poor measure
Somewhat accurate measure
Excellent measure
Not applicable

0 10 20 30 40 50 60 70 80 90 100

How well do typical impact measures reflect real impact?

If you were assigned the task of designing a new way to measure research impact in nursing, how would you measure it? What scale or metric would you create to measure the impact of a specific research or practice article?

Is there anything else you would like to say regarding your perception of “impact factor” or any other scholarship metrics and how they are used to measure the relative importance of scholarly work in a discipline? There is absolutely no expectation for your answer to this question. The purpose of the question is to learn how you view research impact and respond to questions or concerns you might have in the Scholarship Circle course content.

Current stage of writing

Do you have a writing project (or projects) currently in progress?
Yes
No

Regarding your current writing status (not considering past publishing history), what stage would you say your current writing work is in right now? Choose the answer that most closely describes your current writing project(s). You may choose more than one, if you have more than one project in progress now.
Brainstorming, considering interesting topics
Revising the manuscript
Topic is chosen; narrowing down research questions, theoretical frameworks, or approaches to the topic
In the submission process
Reviewing the literature
None of the above
I do not intend to write or finish a manuscript as a result of my participation in the Scholarship Circle
Writing the body of the manuscript

Journal of the Medical Library Association

Supplemental content to
J Med Libr Assoc. Jan;108(1):dx.doi.org/10.5195/jmla.2020.685
www.jmla.mlanet.org
© Dhakal, Tornwall 2020

How many writing projects would you estimate you have in progress right now?

Barrier block

What are the barriers that prevent you from writing and publishing your work?

Limited time to write and publish an article
Limited experience with writing or publishing an article
Lack of motivation or self-discipline
Not knowing how to get started
Lack of incentive related to employment
I have not yet encountered any barriers or challenges
Other

Purpose for attending the Scholarship Circle

What do you hope you will gain from this Scholarship Circle writing experience?

Strategies for finding time to write and publish an article
Experience with strategies and tools for writing and publishing an article
New concepts to help me think about writing or publishing in the future
A manuscript ready to send to a journal by the end of the Scholarship Circle experience
Motivation and strategies for maintaining self-discipline to stay engaged in the writing process
Other

Do you have any other thoughts you would like to share?

End of survey
